# Address at the Twenty-Fourth Annual Commencement of the Ohio College of Dental Surgery

**Published:** 1870-03

**Authors:** W. H. Morgan

**Affiliations:** Nashville, Tennessee


### ADDRESS AT THE TWENTY-FOURTH ANNUAL COMMENCEMENT OF THE OHIO COLLEGE OF DENTAL SURGERY.

BY W. H. MORGAN, M.D., D.D.S., NASHVILLE, TENNESSEE.

*Ladies and Gentlemen:*

In response to the invitation of the faculty of this institution, I am before you this evening to take some humble part in this, its twenty-fourth annual commencement. It is not without some hesitation that I attempt to address you, remembering with what benefit the graduates and the profession, and with what pleasure the audience, generally, have listened to the valedictory on former occasions. I regret that I am not able to offer you a better entertainment to-night, and that one of more ability and more accustomed to public speaking than I, was not selected for this duty.

I feel, however, that no one present will expect from me the ornamental, ornate and finished touches of the experienced orator or essayist.

A few words of advice and encouragement to you, gentlemen of the graduating class, is all that is expected of me, or that I shall attempt. Permit me to congratulate you on the completion of your course, and to express to you the high satisfaction that I, in common with the other Trustees of this college, feel at the highly creditable manner in which you have passed your examination and earned your degrees. Let me assure you that the qualifications necessary to bear your titles honorably, is a guerdon worthy the noblest ambition. To-day marks an era in your lives; it adds another cycle to the past; it is a sort of New Year's day in the calendar of your professional life.

You will readily infer from this that your education is not complete, but that it is just begun in earnest; you are really but just initiated into the way you are to pursue.

You have just reached the first resting-place in your journey up the rugged hill, upon which stands the temple of science, to which there leads no royal road. Look out around you, how much wider is the horizon—how vastly broader the unexplored field? Like the traveler up the mountain-side, each successive step widens the field of vision; and when he shall have gained the topmost pinnacle within mortal reach, he will look out upon the vast untenanted, unexplored, untrodden beyond.

You enter a profession that is still in its infancy, notwithstanding all the boasts you have heard about progress in Dental science. You are young and sanguine; there is much for you to do; we expect much from you. To you, and those of like age, are soon to be entrusted our calling, with all its momentous interests. We exhort you to guard well the sacred trust.

The entire field of science is open before you, inviting you to investigation; and as our knowledge of no science is either complete or full, and as no science is unique or independent, but each one is made up of parts of the other; and knowing, further, that no one man can master all, it would be well for each one of you to select some one of the collateral sciences and make a specialty of it, in connection with Dentistry. Does astronomy attract you? then with telescope go and study the heavenly bodies; tell us of the relations they sustain to each other; their influence upon the earth, the dynamic condition of the atmosphere upon which we are dependent and in which we live; and how through these, the physiological and pathological conditions with which we have to deal, are affected or influenced. Does microscopy invite you, then, with instrument in hand, look down into the infinitesimal world? Tell us its wonders; how the ultimate particles of matter are shaped, and what are their physical relations to each other, and the thousand kindred wonders to be seen there.

The great object of all human effort is happiness. To be happy we must be useful. Our attainments in the former will be in the ratio of our success in the latter. To prepare you for usefulness, by qualifying you for your chosen calling, has been the constant, unselfish effort of your teachers during the past session of this institution. How faithfully they have labored for this result, you can testify; how well they have succeeded, the honors conferred on you this evening, and the future, must determine.

One of the first and highest duties of your new relationship, is to cultivate and cherish a high regard for your profession, bearing in mind this great truth: that the man who understands his calling, who under-estimates its intrinsic value, is unfit for its pursuit—is unworthy a place in its ranks. Such an one is not likely to have a proper regard for himself, and is sure to bring contempt upon his undertakings.

"Forsake not the assembling of yourselves together," is a maxim as true in Dentistry as in religion.

In the olden time, says mythology, Jupiter commanded all the people to assemble annually, bringing with them the proceeds of the last year's labor, and each to explain to the assembly the secret of his success, or apologize for his failure. What was a fable in that dim age is real in our time. A greater than Jupiter calls these assemblies. He who loves and pities stricken humanity looks down and smiles upon all such gatherings, because their efforts are directed to the amelioration of the ills to which flesh is heir.

The great object of every man who enters a Dental Association, should be either to present or receive some truth, and he should be willing to be crucified for its sake; should be willing to be, as it were, brayed in a mortar, that he might by friction, attrition and filtration, be separated from error, so that he may return to his office and patients an humbler and better man—a wiser and more prudent Dentist.

Let every one, of what he has, add something to the common stock of knowledge. Freely you have received; freely give.

As a profession we have, to some extent, compassed earth and sea and air to promote our ends. Every collateral science has been subsidized to our interests; and by labor and merit we have won, and been awarded a place among the liberal professions. And yet we are but a part of what, as a whole, was unworthy the name of profession a few years ago.

A few years since—only a few in the life world—a convention of Castilian physicians and barbers gravely considered, for four days, the propriety of admitting any one to a seat in their body who was only a surgeon.

Who have rescued our calling from opprobrium? Men of our own country; men who were an honor to the age in which they lived, many of them of our own time, some of whom are present with us this evening.

The charlatan and impiric will knock at the door of our temple, and gain admittance, too; why should they not? they seem to be needful. To sharpen the line between the pretender and the true man, and, like many other seemingly necessary evils, are overruled by Providence for good.

No profession has greater need of public confidence than ours. How to get and hold that confidence is one of the important questions you will soon be called upon to solve. A perfect character is what you will want. The nearer you follow the example of Him who went about doing good—whose character was perfection—the more you will be esteemed, at least by the good, and the more readily you will get hold upon public favor. Fix a perfect model in your mind, and then mould yourself to it.

Society demands that the Dentist shall be a gentleman of the first mark; the demand is unequivocal, justly so. The mothers, wives and daughters of the age are entrusted to our care. Ours is a genteel profession. We have less temptation, and so less excuse, for dissipation than any other—save the holy ministry of the Gospel. The lawyer, in his anxious wrangle, has often to contend with the false witness and unjust judge. The physician has his stormy rides, his sleepless nights and pennyless patients.

Sunset closes our labors; when, with slippers and gown, we are allowed the joys and happiness of home, in which to cultivate our better impulses and refine our natures. Your quiet evenings will afford you opportunity for study and improvement, not only in your profession, but in the polite literature of the past and present. You will be called upon for the most part to serve the cultivated and refined. See to it that you are qualified for such association. Ladies, what shall I say to you, or of you. A man like myself, who has no poetry in his nature, or music in his soul, scarcely knows how to address your sex. Your presence here shows your appreciation of our efforts in behalf of suffering humanity.

Shall I not urge these young gentlemen to seek the refining influence of female society, and say to them that every man is a failure who does not?

Where shall I locate? is a question you have often asked yourselves recently. In making your selection, seek a locality where you can readily put yourself in sympathy with the people, and your professional brethren who may be near you, one that affords at least reasonable prospects of success. That is business. "Birds of a feather should flock together." This, like all other laws of nature, is founded on immutable principle. Therefore, seek a place among those of like feelings and views with yourselves. Let your office arrangements be neat and cleanly, elegant and attractive when you have the means to afford it. Gather around you all the evidences of refinement compatible with your circumstances.

True merit will always give a just renown. The reward may seem to delay, but it will come at last. Remember sunset is the hour for wages. You may expect and have a right to demand time and means for refreshment and rest, but the hour to receive wages as the reward of your works, is at the close of the day of life, when its toils are over and its labors are accomplished.

Gentlemen, my task is done; and now you go forth in the world where you choose, with Providence your guide. Hoping that your brightest anticipations may be realized, your highest aspirations attained, and the just expectations of your *Alma Mater* fulfilled, and that you may, when life's toil is over, find an eternal resting-place in the "flowery land beyond," I, in the name of the Faculty of the Ohio College of Dental Surgery, bid you an affectionate farewell.

